# *Rpl3l* gene deletion in mice reduces heart weight over time

**DOI:** 10.3389/fphys.2023.1054169

**Published:** 2023-01-17

**Authors:** Kelly M. Grimes, Vikram Prasad, Jiuzhou Huo, Yasuhide Kuwabara, Davy Vanhoutte, Tanya A. Baldwin, Stephanie L. K. Bowers, Anne Katrine Z. Johansen, Michelle A. Sargent, Suh-Chin J. Lin, Jeffery D. Molkentin

**Affiliations:** Department of Pediatrics, Cincinnati Children’s Hospital Medical Center, University of Cincinnati, Cincinnati, OH, United States

**Keywords:** heart, ribosome, translation, hypertrophy, gene expression

## Abstract

**Introduction:** The ribosomal protein L3-like (RPL3L) is a heart and skeletal muscle-specific ribosomal protein and paralogue of the more ubiquitously expressed RPL3 protein. Mutations in the human *RPL3L* gene are linked to childhood cardiomyopathy and age-related atrial fibrillation, yet the function of RPL3L in the mammalian heart remains unknown.

**Methods and Results:** Here, we observed that mouse cardiac ventricles express RPL3 at birth, where it is gradually replaced by RPL3L in adulthood but re-expressed with induction of hypertrophy in adults. *Rpl3l* gene-deleted mice were generated to examine the role of this gene in the heart, although *Rpl3l* ^*−/−*^ mice showed no overt changes in cardiac structure or function at baseline or after pressure overload hypertrophy, likely because RPL3 expression was upregulated and maintained in adulthood. mRNA expression analysis and ribosome profiling failed to show differences between the hearts of *Rpl3l* null and wild type mice in adulthood. Moreover, ribosomes lacking RPL3L showed no differences in localization within cardiomyocytes compared to wild type controls, nor was there an alteration in cardiac tissue ultrastructure or mitochondrial function in adult *Rpl3l* ^*−/−*^ mice. Similarly, overexpression of either RPL3 or RPL3L with adeno-associated virus −9 in the hearts of mice did not cause discernable pathology. However, by 18 months of age *Rpl3l* ^*−/−*^ null mice had significantly smaller hearts compared to wild type littermates.

**Conclusion:** Thus, deletion of *Rpl3l* forces maintenance of RPL3 expression within the heart that appears to fully compensate for the loss of RPL3L, although older *Rpl3l* ^−/−^ mice showed a mild but significant reduction in heart weight.

## Introduction

Because levels of mRNA translation are relatively low in the adult mammalian heart and cardiomyocytes are refractory to proliferation (Chorghade et al., 2017), it stands to reason that cardiomyocytes may have evolved different mechanisms of translational control given that they largely persist for the life of the organism. Indeed, heart and skeletal muscle uniquely express the ribosomal protein L3-like (RPL3L), which is a paralog of the RPL3 gene that is expressed from mammals to yeast (Rosado et al., 2007; Malecki et al., 2021). The existence of these paralogs suggests that such gene pairs may have evolved to mediate ribosomal control of translation across tissues with highly specialized properties, such as striated muscle (Gerst, 2018). Eukaryotic 80 S ribosomes are composed of small (40S) and large (60S) subunits, in which approximately 80 ribosomal proteins surround four core ribosomal RNAs (rRNAs) (Yusupova and Yusupov, 2016). While there are many invariant proteins, in some instances ribosomal proteins have undergone gene duplication and exist in paralog pairs, such as RPL3 and RPL3L, and this heterogeneity is thought to help confer translational specificity (Gerst, 2018). From studies of yeast ribosomes, RPL3 expression is critical for 60S ribosomal subunit assembly, and ablation of this gene results in malformed polysomes and halting of cell cycle in the G1 phase (Rosado et al., 2007). Also demonstrated in yeast, the RPL3 protein sits close to the peptidyl transferase center, and x-ray crystallography has determined that its incorporation into the ribosome is critical for its proper organization and function such that mutations in this gene can dramatically alter translation (Mailliot et al., 2016). In contrast, there exists much less information on its paralog, RPL3L, which has evolved later down the phylogenic tree and is only expressed in heart and skeletal muscle (Van Raay et al., 1996).

*RPL3L* appears to play a crucial role in human cardiac function as mutations in this gene are associated with aging-associated atrial fibrillation (Thorolfsdottir et al., 2018) and lethal neonatal cardiac dilation (Ganapathi et al., 2020; Das et al., 2022; Nannapaneni et al., 2022). Another recent case study discovered a heterozygous *RPL3L* genetic variant in children with catecholaminergic polymorphic ventricular tachycardia (Jaouadi et al., 2022). Here, we have generated a mouse with *Rpl3l* gene deletion to examine the function of this striated-muscle specific ribosomal paralog. We hypothesized that *Rpl3l* ^−/−^ mice would have an overt phenotype given the unique contractile function of the cells in which it is expressed. Intriguingly, loss of RPL3L protein is fully compensated by re-expression of RPL3 protein in mouse heart and skeletal muscle and no overt phenotype was identified until 18 months of age when a subtle but significant reduction in heart weight was observed.

## Results

**FIGURE 1:**
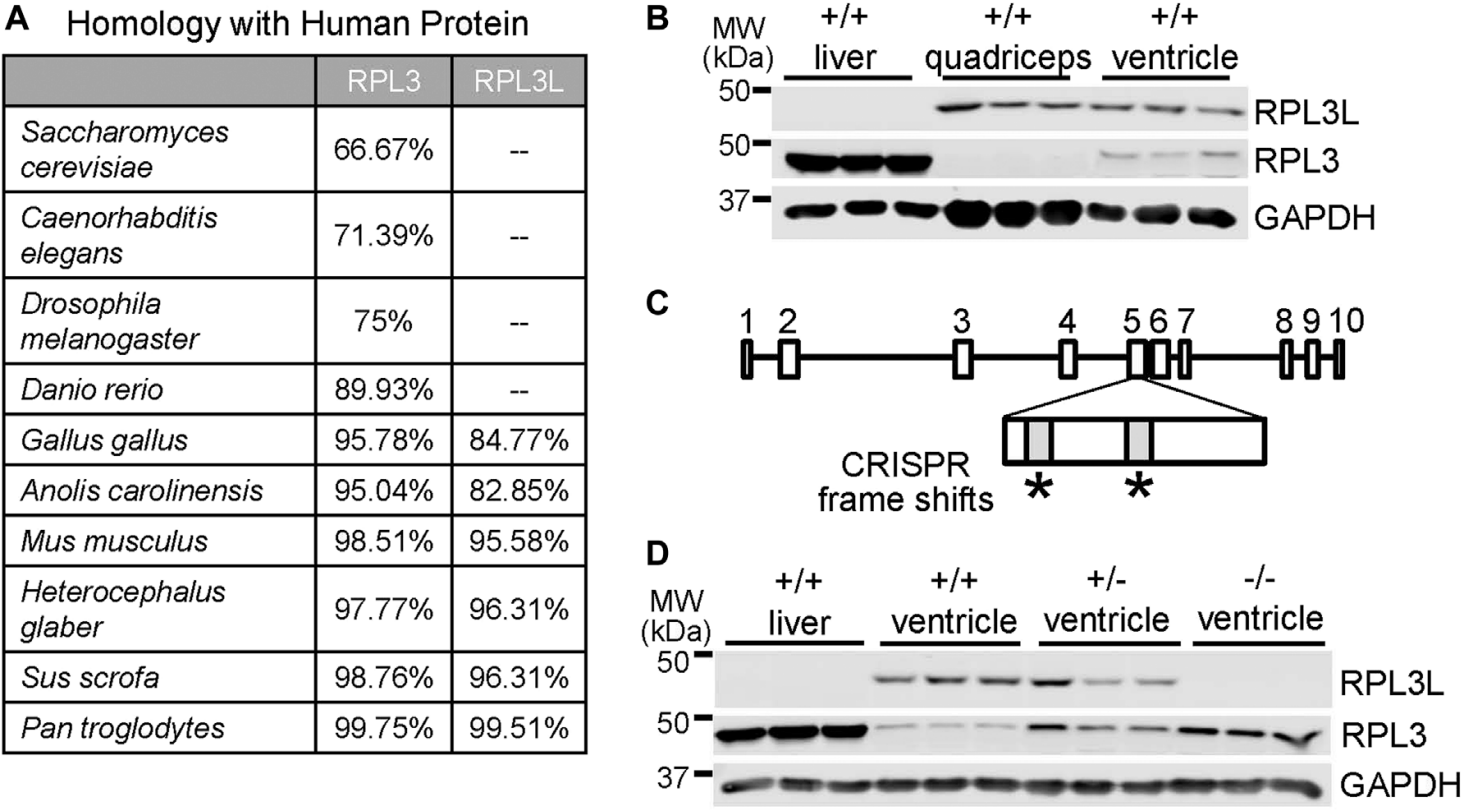
Generation of *Rpl3l* gene-deleted mice. **(A)** Table showing evolutionary amino acid sequence conservation as percentage identity for human RPL3L and RPL3 generated from sequences in the NCBI database. **(B)** Western blot showing RPL3L versus RPL3 in adult liver, quadriceps skeletal muscle and heart ventricles at 2 months of age. GAPDH was used as a control for tissue processing and blotting (*n* = 3 in each group). MW denotes molecular weight in kilodaltons (kDa). **(C)** Schematic of the mouse *Rpl3l* gene locus and CRISPR strategy utilized to target two areas of exon five to induce a frameshift and subsequent deletion mutation. **(D)** Western blotting of RPL3L and RPL3 from liver or cardiac ventricles of wild type (+/+), *Rpl3l* ^*+/−*^ and *Rpl3l* ^*−/−*^ mice, confirming loss of RPL3L protein in the hearts of the −/− (nullizygous) CRISPR targeted mice.

While RPL3 is conserved throughout animal, plant, and fungi evolution, its recently duplicated paralogue RPL3L is first observed across phylogeny in avians and mammals. Homology scores between RPL3 and RPL3L were generated by comparing National Center for Biotechnology Information (NCBI) sequences to the human proteins (Figure 1A). Rodents, pigs, and chimpanzees all have a high degree of sequence similarity in both paralogs compared to the human versions of the proteins. Yeast, invertebrates, reptiles and most fish only express RPL3, although the thorny skate fish appears to be unique in also having the *Rpl3l* gene. A unique feature of avians and mammals compared with reptiles and fish is a more highly specialized and complex heart and skeletal muscle cytoarchitecture for optimal contractile activity. To examine the characteristics and potential functional role of the *Rpl3l* gene in the mouse heart we generated custom antibodies due to issues with commercially available products. To generate these antibodies, we used PCR to confirm that *Rpl3l* mRNA present in the mouse heart was exclusively one spliced form (transcript variant 1, out of the two that are annotated), which corresponded with the highly conserved amino acid sequence previously reported (see methods). Expression of RPL3 and RPL3L determined by western blotting varies across adult wild type mouse tissues, such that liver expresses only RPL3, quadriceps muscle tissue expresses only RPL3L, and adult heart tissue expresses a combination of both but a predominance of RPL3L (Figure 1B).

Next, we generated CRISPR-targeted mice in which two frame-shift mutations were introduced into the 5th exon of the *Rpl3l* gene (Figure 1C), which generated a null allele for protein expression as confirmed by western blotting from adult tissues (Figure 1D). Interestingly, RPL3L protein expression was fully compensated by RPL3 expression, likely explaining why these mice were viable. Indeed, western blots showed upregulated RPL3 protein in the ventricles of adult mouse hearts in both *Rpl3l* ^*+/−*^ (heterozygotes) and *Rpl3l* ^*−/−*^ mice (Figure 1D). Quantitative PCR also showed a decrease in *Rpl3l* mRNA from the hearts of these gene-deleted mice (Supplementary Figure S1). Interestingly, quadriceps from adult *Rpl3l* ^*−/−*^ mice showed induction of RPL3 expression, which is noteworthy because RPL3 protein is not normally expressed in adult quadriceps muscle (Supplementary Figure S2A), underscoring the observed compensation between these two paralogue genes in striated muscle. However, the fact that mutations in the human *RPL3L* gene can result in early neonatal lethality, severe cardiomyopathy, and arrhythmia (Ganapathi et al., 2020; Das et al., 2022; Nannapaneni et al., 2022) while *Rpl3l* ^*−/−*^ mice appear overtly normal as young adults may reflect a lack of compensation by RPL3 in humans or more likely that mutant RPL3L human protein remains fully intact (hence not giving a signal to upregulate RPL3) and is fully incorporated within the 60S ribosome, which causes translational dysfunction.

**FIGURE 2:**
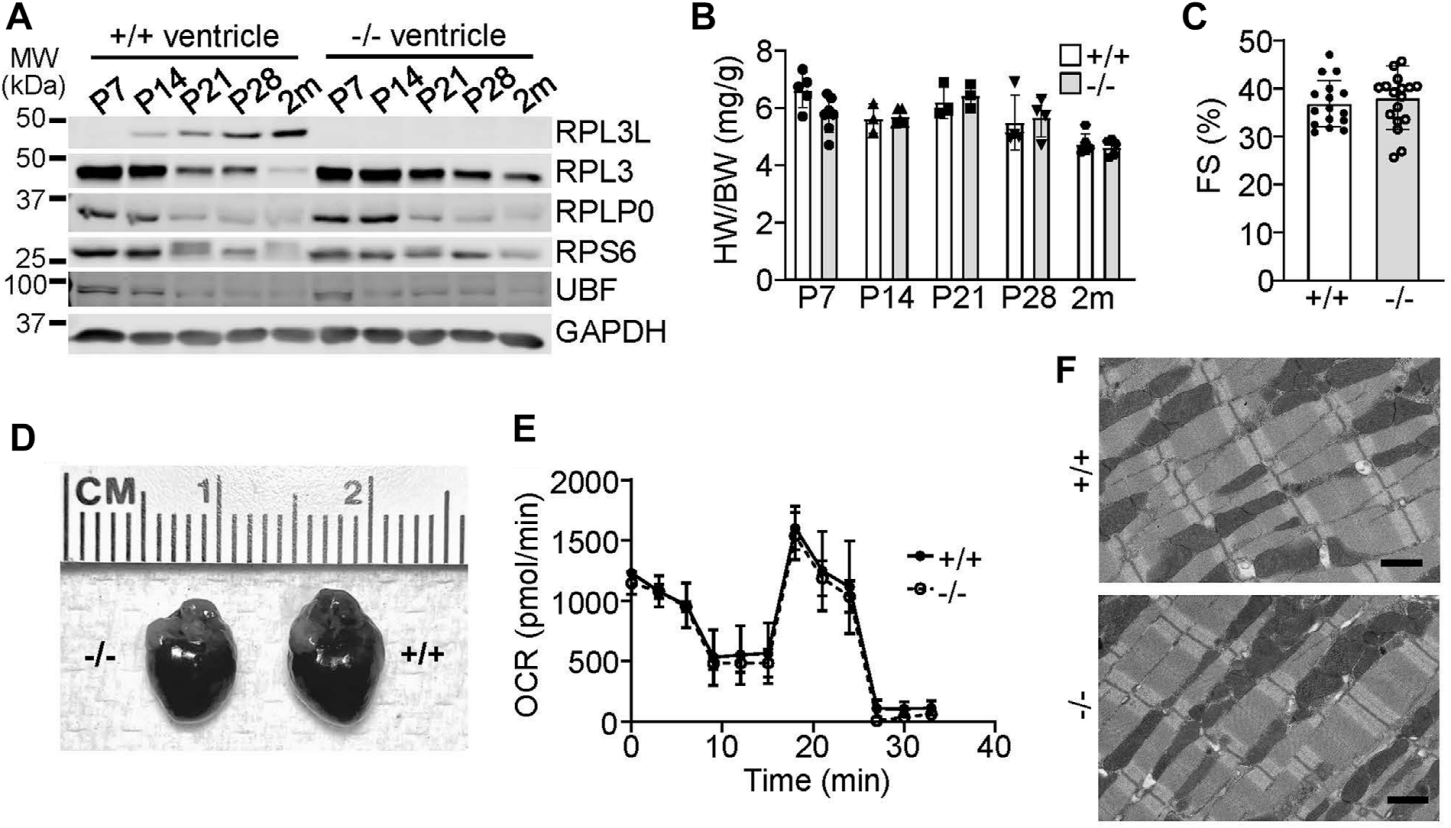
RPL3L and RPL3 protein expression switch during development in the mouse heart. **(A)** Western blotting for the indicated proteins at the indicated times after birth from hearts of wild type (+/+) or *Rpl3l* ^*−/−*^ (−/−) mouse ventricles. GAPDH is used as a processing and loading control. MW denotes molecular weight in kilodaltons (kDa). **(B)** Heart weight to body weight ratios in wild type (+/+) or *Rpl3l* ^*−/−*^ (−/−) mice over the indicated developmental time points (*n* = 3–7 per group). **(C)** Fractional shortening (FS) measured by echocardiography at 2 months of age in wild type and *Rpl3l* ^*−/−*^ (*n* = 16–17). **(D)** Gross morphological images of hearts from wild type or *Rpl3l* ^*−/−*^ mice at 2 months of age. **(E)** Oxygen consumption rate (OCR) of isolated cardiac mitochondria from hearts of the indicated two groups of mice at 2 months of age (*n* = 3). The OCR experiment extended over time (min) with sequential addition of respiratory chain complex inhibitors at the different inflection points in the curves shown. **(F)** Electron micrograph showing sarcomeric and mitochondrial ultrastructure in heart histological sections in 2-month-old mice (scale bars = 1 µm). All data are represented as mean ± SEM.

We examined the expression characteristics of RPL3 and RPL3L more carefully in the heart, which showed absence of RPL3L protein in the mouse heart up to 7 days after birth, at which time only RPL3 was highly expressed (Figure 2A). By 28 days of age cardiac ribosomes showed equivalent expression of both paralogs, and then by 2 months of age RPL3L became the dominant protein paralog expressed in the mouse heart (Figure 2A). Importantly, the expression of other ribosomal subunit proteins, such as RPLP0 and RPS6, were not changed in the hearts of *Rpl3l* ^*−/−*^ mice compared with wild type mice, nor was expression of the upstream binding factor (UBF) protein, which is a master regulator of ribosomal gene transcription (Figure 2A).

With respect to a cardiac phenotype, heart size was similar between the wild type and *Rpl3l* ^*−/−*^ mice at all points examined in postnatal development (Figure 2B). In 2-month-old mice there was no difference in cardiac ventricular performance as measured by echocardiography (Figure 2C) or in gross morphology (Figure 2D). While a recent preprint reported that RPL3L can impact mitochondrial function (Milenkovic et al., 2021), metabolic output of isolated cardiac mitochondria was similar between wild type versus *Rpl3l* ^*−/−*^ mice (Figure 2E). Furthermore, electron microscopy of hearts of *Rpl3l* ^*−/−*^ mice at 2 months of age showed no ultrastructural abnormalities compared with wild type mice, including mitochondria (Figure 2F). Similarly, we subjected *Rpl3l* ^*−/−*^ mice to treadmill running protocols as a measure of their cardiovascular and skeletal muscle fitness, which showed no difference in performance between the two genotypes (Supplementary Figure S2B). Tibialis anterior skeletal muscle weights at baseline and following 10 days of denervation were also similar in absolute mass between the two genotypes, suggesting that loss of *Rpl3l* did not impact skeletal muscle growth characteristics or its atrophy with denervation (Supplementary Figure S2C).

**FIGURE 3:**
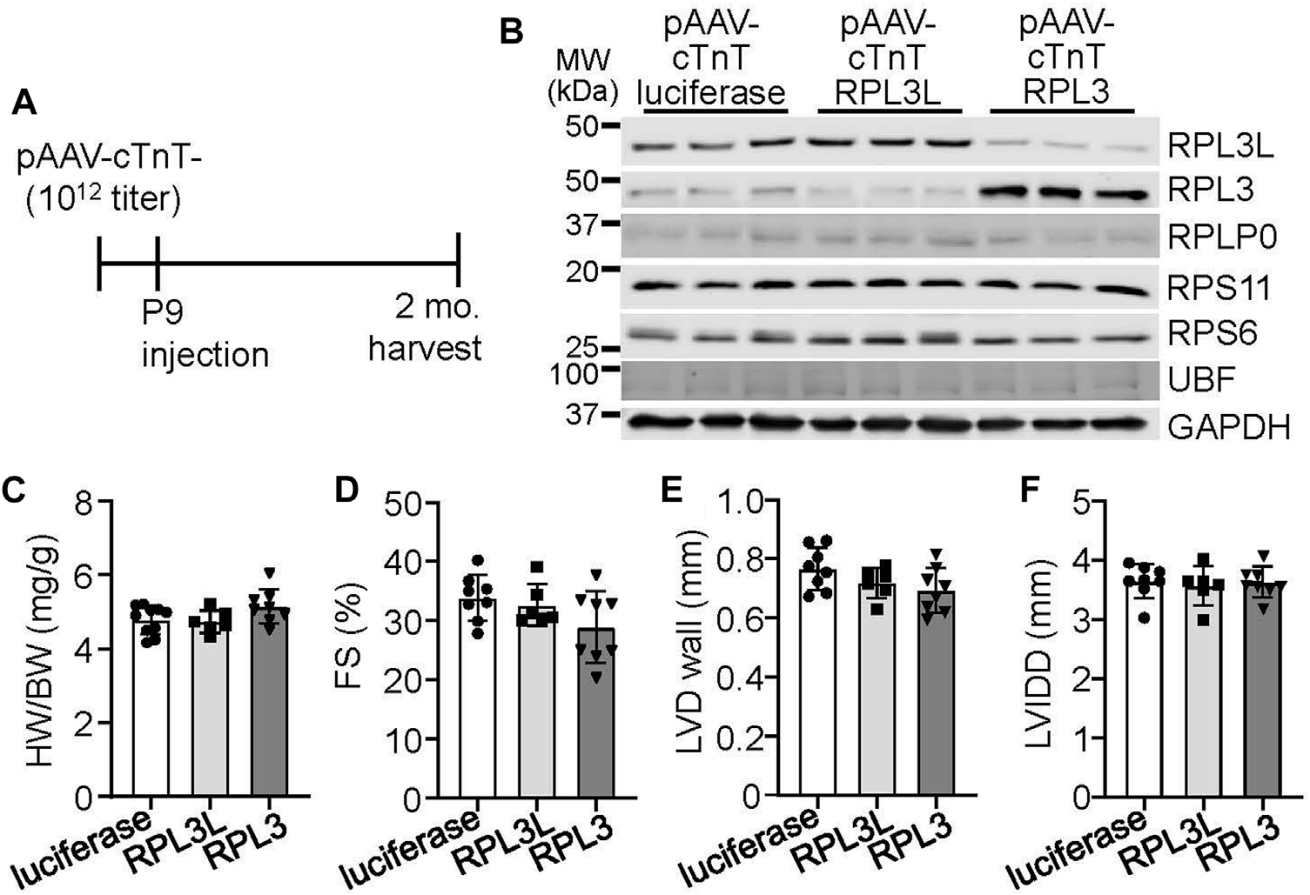
Adeno associated virus 9 (AAV9)-mediated overexpression of RPL3L and RPL3 does not alter heart structure or function. **(A)** Schematic showing timeline of intrathoracic injections of the recombinant AAVs encoding luciferase, RPL3L, and RPL3 into P9 pups at a titer of 1 × 10^12^ genome copies followed by harvest and analysis at 2 months of age. **(B)** Western blots of the indicated proteins in cardiac ventricles from mice injected with AAV-luciferase, -RPL3L, and -RPL3. GAPDH is used is a loading control (*n* = 3 per group). MW denotes molecular weight in kilodaltons (kDa). **(C–F)** Heart weight-to-body weight ratio (HW/BW), fractional shortening (FS), average left ventricular wall thickness in diastole (LVD wall) and left ventricular internal dimension in diastole (LVIDD) in mice injected with the indicated AAVs as shown (*n* = 6–10 per group). Data are mean ± SEM. No statistical differences between the groups of mice were observed.

As both RPL3 and RPL3L are expressed in adult mouse ventricles, we sought to determine if the overexpression of either would affect cardiac physiology by utilizing adeno-associated virus-9 (AAV9) targeted to cardiomyocytes with a cardiac troponin T promoter (Figure 3A). Overexpression of either paralog drove the protein expression of the other protein down, again showing that they exist in a stoichiometric relationship to one another by reciprocal placement within the ribosome where they achieve long-term stability (Figure 3B). However, overexpression of either protein did not cause functional differences or changes in cardiac morphology and ventricular performance by 2 months of age (Figures 3C–F). An AAV9-luciferase construct was used as a control for these experiments.

**FIGURE 4:**
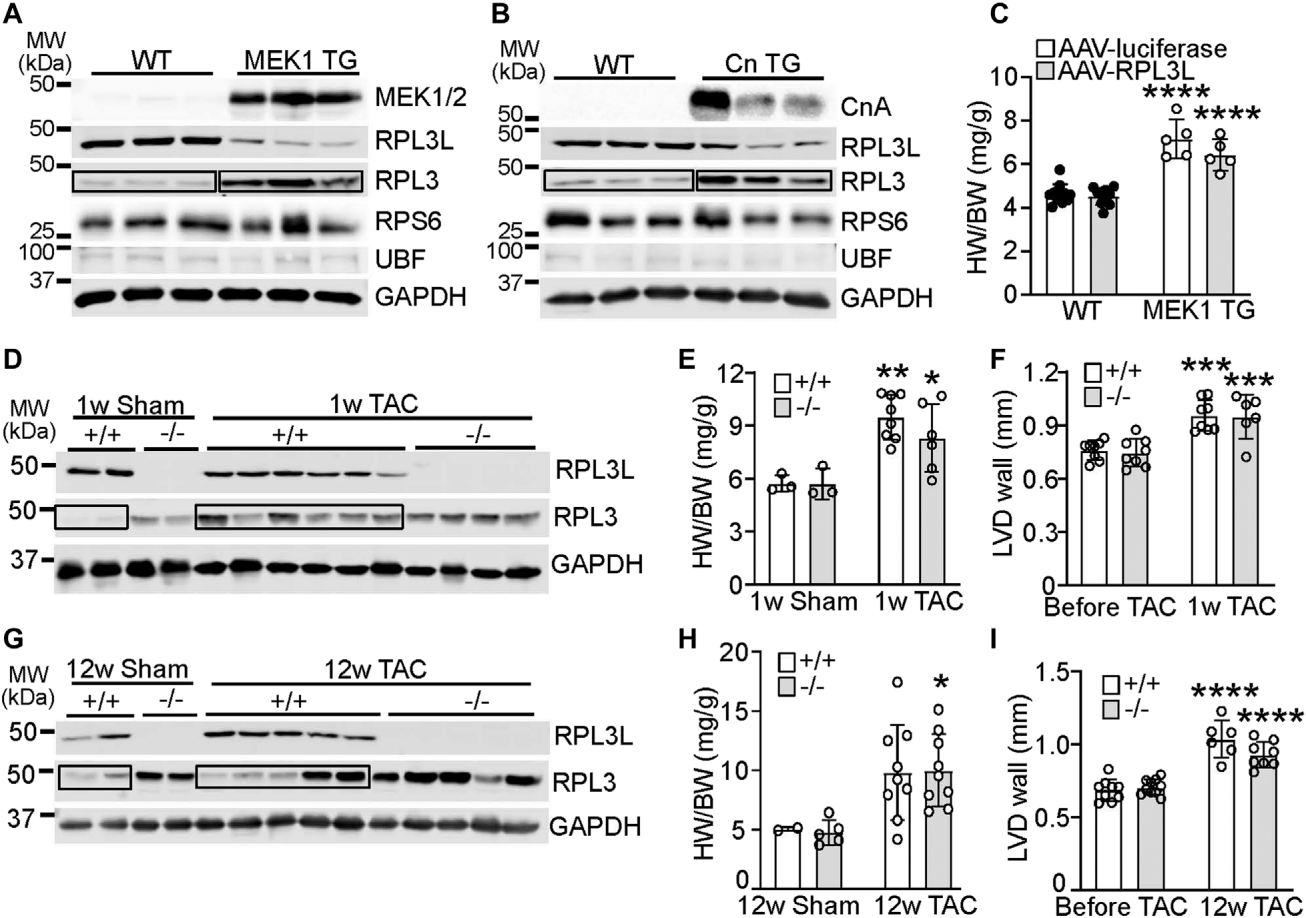
RPL3 and RPL3L paralog switching occurs during active hypertrophy but is not causative. **(A)** Western blots showing expression of the indicated proteins in hearts from transgenic mice expressing activated MEK1 compared to wild type littermates (*n* = 3). MW denotes molecular weight in kilodaltons (kDa). The boxed area shows the upregulation of RPL3 protein in the TG hearts versus the control. **(B)** Western blots showing expression of the indicated proteins in hearts from transgenic mice expressing activated calcineurin (CnTG) compared to wild type littermates (*n* = 3). CnA represents the migration of the activated form of the calcineurin A protein from the Cn transgene, which is shorter than the endogenous form of calcineurin. The boxed area shows the upregulation of RPL3 protein in the TG hearts versus control. **(C)** Heart weight to body weight ratios in 3-month-old MEK1 transgenic and wild type mice previously injected with AAV-luciferase or AAV-RPL3L (*n* = 5–11, **** = *p* < 0.0001 compared to respective wild type controls, by Sidak’s multiple comparisons test after 2-way ANOVA). **(D)** Western blotting for RPL3 and RPL3L expression in wild type and *Rpl3l* ^−/−^ hearts after TAC or a sham surgical procedure begun at 2 months of age and followed for 1 week before harvest. GAPDH is shown as a processing and loading control. (*n* = 2–6/group). The boxed area shows the increase in RPL3 protein with TAC in the +/+ (WT) mouse hearts compared to respective sham. **(E)** HW/BW ratios of wild type and *Rpl3l* ^−/−^ mice after TAC or sham procedure for 1 week (*n* = 3–8, ** = *p* < 0.01, * = *p* < 0.05 compared to respective sham controls, by Sidak’s multiple comparisons test after 2-way ANOVA). **(F)** Average left ventricular wall thickness by echocardiography in diastole (LVD wall) of wild type and *Rpl3l* ^*−/−*^ mice at baseline and 1 week after TAC surgery (*n* = 6–8; *** = *p* < 0.001 compared to same mice before TAC, by Sidak’s multiple comparisons test after 2-way ANOVA). **(G)** Western blotting for RPL3 and RPL3L expression in wild type and *Rpl3l* ^−/−^ hearts after TAC compared to a sham surgical procedure begun at 2 months of age and followed for 12 weeks before harvest (*n* = 2–5/group). GAPDH is shown as a processing and loading control. The boxed areas show the changes in RPL3 protein with TAC in the +/+ (WT) mouse hearts compared to sham. **(H)** HW/BW ratios of wild type and *Rpl3l* ^−/−^ after TAC or sham procedure for 12 weeks (*n* = 2–9, * = *p* < 0.05 compared to respective sham controls, by Sidak’s multiple comparisons test after 2-way ANOVA). **(I)** Average left ventricular wall thickness in diastole (LVD wall) of mice at baseline and 12 weeks after TAC surgery (*n* = 6–12; **** = *p* < 0.0001 compared to same mice before TAC, by Sidak’s multiple comparisons test after 2-way ANOVA). Data are mean ± SEM.

In cardiac biology it is not uncommon for paralog genes that switch during development to show reversion to the fetal or neonatal state with induction of cardiac disease or hypertrophy (Taegtmeyer et al., 2010). Indeed, transgenic mice overexpressing an activated form of mitogen-activated protein kinase kinase (MEK1; Figure 4A) or a constitutively active form of calcineurin (CnTG: Figure 4B), both of which exhibit significant cardiac hypertrophy by 3 months of age, showed prominent re-expression of the RPL3 protein and loss of RPL3L in the heart (Figures 4A, B). Importantly there was no change in expression of RPS6 or UBF in either of these mouse lines (Figures 4A, B), suggesting there was no change in ribosome number. Moreover, forcing maintenance of RPL3L expression within the MEK1 transgenic hearts with AAV9 did not alter the hypertrophic response that normally characterizes this model (Figure 4 C). This result again suggests that RPL3L is not directly impacting mRNA translation within the heart in a manner that would alter the cardiac hypertrophic response. Intriguingly, cardiac hypertrophy induced by transverse aortic constriction (TAC) in 2-month-old mice for 1 week resulted in a similar degree of heart growth and increase in ventricular wall thickness between *Rpl3l* ^*−/−*^ and wild type mice; however, wild type mice subjected to TAC exhibited re-expression of the RPL3 protein and loss of RPL3L, similar to the transgenic models of hypertrophy (Figures 4D–F). There was also similar amounts of cardiac hypertrophy and ventricular wall thickness increase between the groups of mice 12 weeks after TAC surgery (Figures 4H, I). However, the re-expression of RPL3 was somewhat more variable at 12 weeks of TAC likely due to variation in the degree of heart failure in each of these mice (Figure 4G). For full echocardiography parameters, refer to Supplementary Tables S2, S3.

**FIGURE 5:**
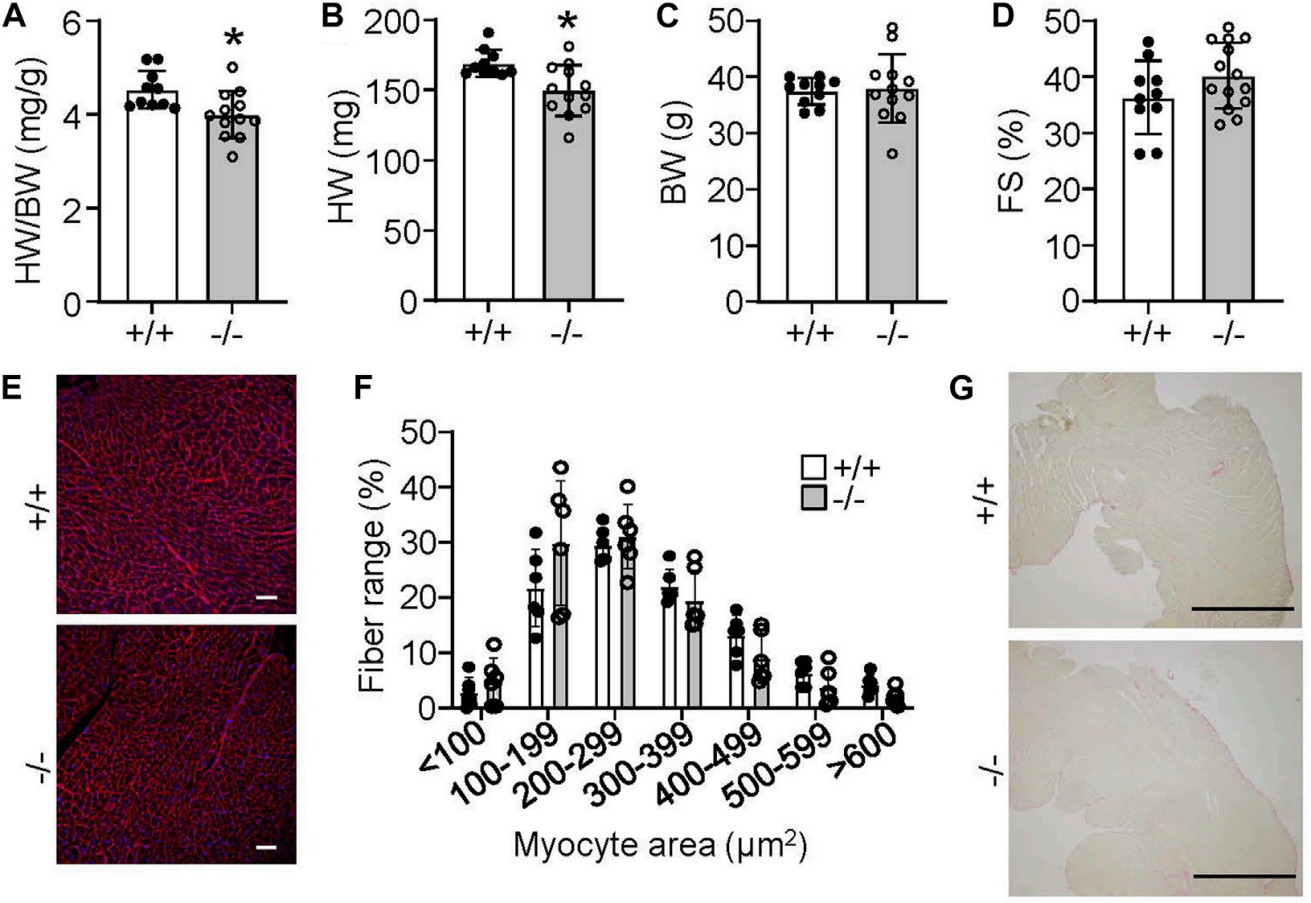
*Rpl3l* ^*−/−*^ mice have reduced heart size at 18 months of age. **(A–C)** HW/BW ratio, absolute HW and absolute BW in *Rpl3l* ^−/−^ (−/−) and wild type (+/+) mice at 18 months of age (*n* = 10–12, * = *p* < 0.01. **(D)** Fractional shortening (FS) measured by echocardiography at 18 months of age in the two indicated groups of mice (*n* = 10–14). **(E,F)** Microscopic images of cardiac histological sections stained with wheat germ agglutinin (WGA)-Alexa fluor 594 conjugate in red **(E)** and quantification **(F)** of cardiomyocyte cross-sectional area between wild type and *Rpl3l* ^*−/−*^ mice at 18 months of age from the histological images (scale bars = 100 µm). **(G)** Microscopic images of cardiac histological sections stained with picrosirius red to show collagen deposition between the two groups of mice at 18 months of age (scale bars = 1 mm). Data are mean ± SEM.

Despite the lack of an overt phenotype in *Rpl3l* ^*−/−*^ mice through our various analyses of cardiac structure and function, we did uncover an age-related phenotype at 18 months. At this age *Rpl3l* ^−/−^ mice showed significantly smaller hearts compared to their wild type controls, but no change in body weight (Figures 5A–C). Like younger mice, cardiac ventricular performance remained unaffected compared with 18-month-old wild type controls (Figure 5D). Histological examination also did not identify overt differences between aged wild type and *Rpl3l* ^*−/−*^ mice in terms of tissue architecture, cardiomyocyte cross-sectional area, and fibrosis (Figures 5E–G).

**FIGURE 6:**
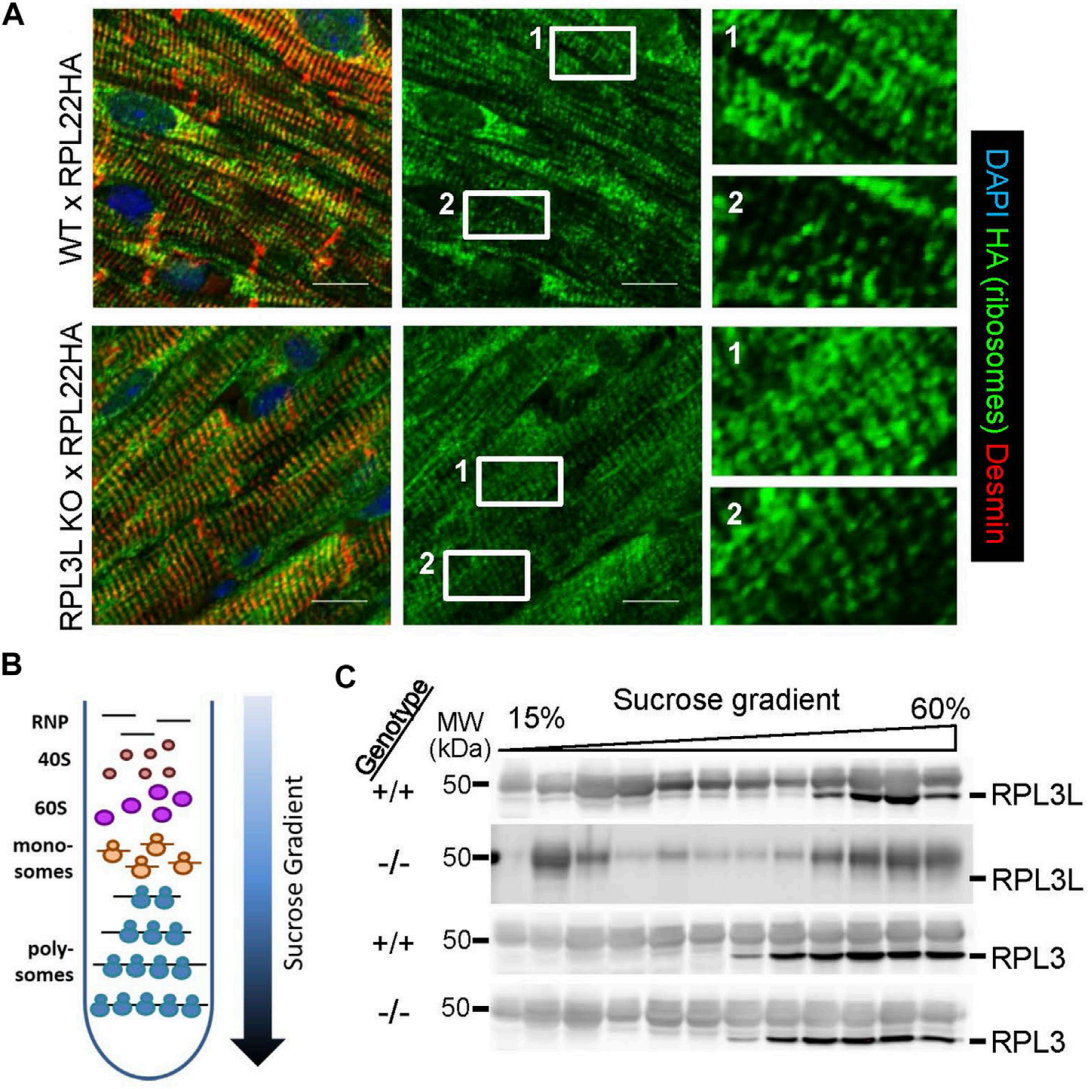
Ribosome localization and polysome fractionation between the genotypes. **(A)** Immunostaining for HA-tagged RPL22 in histological sections from hearts of RiboTag (RPL22-HA) mice crossed with wild type and *Rpl3l* ^−/−^ mice at 2 months of age. The left panels are a merge of HA tag (green) counterstained with desmin (red) and DAPI (blue) to show nuclei, while the center panels show only the HA tag staining (green). The right panels are ×6 magnifications of the noted areas (1 & 2) in the HA stain (green) seen in the middle panels (scale bars = 5 µm). **(B)** Schematic of the sucrose gradients used to fractionate ribosomes into different subpopulations for ribosome profiling analysis of mRNA and western blot analysis of ribosome subunits. **(C)** Western blots of individual fractions from the sucrose gradients showing RPL3 and RPL3L expression across the ribosomal populations in *Rpl3l* ^−/−^ (−/−) or wild type (+/+) hearts. MW denotes molecular weight in kilodaltons (kDa).

We originally hypothesized that sustained RPL3 expression in the heart due to deletion of the *Rpl3l* gene would be linked with greater rates of sarcomerogenesis or hypertrophy. This is in part because RPL3 is dominantly expressed in the heart when it is undergoing hypertrophy during postnatal development and adding new sarcomeres, and because RPL3 was upregulated in expression during hypertrophy. However, by 18 months of age *Rpl3l* ^*−/−*^ mice had significantly smaller hearts suggesting a potentially more complex regulatory role for this paralogue. Hence, we first surveyed ribosome distribution in cardiomyocytes of the heart using RiboTag genetically modified mice, which contain a gene-targeted *Rpl22* construct engineered with a hemagglutinin (HA) protein tag. RiboTag mice were crossed with transgenic mice expressing Cre recombinase from the α-myosin heavy chain promoter (α-MHC-Cre) so that *Rpl22*-HA was specifically recombined to express the tagged protein in cardiomyocytes of the adult heart, which could then be interrogated for ribosome localization. These mice were crossed into the *Rpl3l* ^*−/−*^ or wild type backgrounds and hearts were removed at 2 months of age and subjected to immunohistochemistry for HA staining in cardiomyocytes. Both groups of mice displayed similar distributions of ribosomes (Figure 6A), which appeared both as uniform lines co-localizing with the z-disk, as well as doublets on either side of the z-disk as previously identified for ribosomal patterning in cardiomyocytes (Scarborough et al., 2021).

Analysis of wild type and *Rpl3l* ^*−/−*^ mouse hearts by Affymetrix microarray analysis for alterations in mRNA levels did not identify significant changes in cardiac gene expression pathways, and the handful of genes that were significantly changed were likely due to statistical anomalies that normally occur in these large gene sets (GSE216168). Indeed, qPCR validation of the five most upregulated and down-regulated genes only verified *Rpl3l and Cdkn1a* as altered (Supplementary Figure S1A, B). We then undertook ribosome sequencing from the hearts of these mice to determine if there was a change in the mRNAs preferentially translated by adult cardiac ribosomes lacking RPL3L. Here, we fractionated monosomes and polysomes on a sucrose gradient and combined them for ribosome sequencing (Figure 6B). This yielded only 11 differentially expressed transcripts in hearts between *Rpl3l* ^−/−^ and wild type mice, two of which were un-annotated (GSE215869). We again conducted qPCR validation on cDNA from the ribosome sequencing samples, and out of the nine targets, only *Rpl3l* mRNA was confirmed to have changed (Supplementary Figure S1C, D). Taken together, these results suggest that loss of RPL3L protein does not cause a significant effect on global mRNA levels or mRNAs being actively translated in the hearts of young adult mice. Hence, it is likely that the reduction in heart size observed in *Rpl3l* ^*−/−*^ mice at 18 months of age is the result of subtle changes in ribosomal activity and associated mRNA translation that accumulates over the lifespan of the mouse and is not detectable with acutely designed experimental approaches.

Finally, protein was also purified from individual fractions across the sucrose gradients and probed for RPL3 and RPL3L protein by western blotting. The data show that RPL3 protein is present in the same fractions of the monosomes to polysome gradients whether the heart is wild type or *Rpl3l* ^−/−^(Figure 6C). However, while RPL3L protein is most highly expressed in the polysome-containing fractions, it also appears in the monosome-containing fractions, in contrast to RPL3 that is exclusively found within polysome fractions.

## Discussion

The striking developmental switching between RPL3 and RPL3L proteins in the mouse heart (Figure 2A) follows those of other well-studied fetal-adult protein pairs that switch in the heart, such as α- and β-MHC (Taegtmeyer et al., 2010). Developmentally linked changes in *Rpl3l* and *Rpl3* genes in the mouse heart have also been shown in a recent preprint (Milenkovic et al., 2021). The switching of these paralogs suggests a potential function in cardiac development or maturation and growth of the heart; however, in our hands loss of the *Rpl3l* gene did not alter heart development in the mouse. Interestingly, *Rpl3l* was reported to be one of the most highly downregulated genes in an analysis of adult mouse cardiomyocytes reprogrammed to a fetal state (Chen et al., 2021). Yet another study of maturation markers in human induced pluripotent stem cell cardiomyocytes found that these cells do not express *Rpl3l* mRNA even after 1 year in culture, likely because they never fully differentiate (Anzai et al., 2020).

It remains unclear how *Rpl3* and *Rpl3l* are transcriptionally linked and how loss of RPL3L protein expression in cardiac and skeletal muscle leads to induction of RPL3 in the mouse (Figure 1D; Supplementary Figure S2A, and Affymetrix array data). A recent preprint suggested that reversing expression from RPL3L to RPL3 in adult mouse hearts promotes interactions between ribosomes and mitochondria, thus affecting ribosome localization (Milenkovic et al., 2021). This study also showed upregulation of respiration and mitochondrial genes in hearts lacking *Rpl3l*. However, we did not observe a difference in mitochondrial function or structure between wild type and *Rpl3l* ^−/−^ hearts (Figures 2E, F). Furthermore, both our microarray and ribosome sequencing data sets failed to identify changes in mitochondrial gene expression (Supplementary Figure S1). We also did not detect overt changes in ribosome localization in cardiac myocytes, as ribosomes from both genotypes showed similar staining patterns when crossed with the RiboTag mice (Figure 6A). In humans, missense mutations in *RPL3L* cause cardiomyopathy and lethal arrhythmia (Ganapathi et al., 2020; Das et al., 2022; Nannapaneni et al., 2022), and one of these studies showed no alteration in the patient’s mitochondrial genome due to the RPL3L missense mutation (Das et al., 2022). Given the lack of a cardiac phenotype in *Rpl3l* null mice, it is likely that missense mutations in *RPL3L* from human patients function in a dominant manner by negatively impacting ribosome function, and because RPL3L protein expression is likely unaltered, there is no compensation by RPL3 as we observed in *Rpl3l* ^*−/−*^ mice (see below for discussion of yeast mutant RPL3).

The paralog switching observed in the mouse ventricle has been shown previously in skeletal muscle. Mice subjected to overload-induced hypertrophy of the plantaris muscle showed reduced expression of *Rpl3l* and upregulated *Rpl3* mRNA (Chaillou et al., 2016). Furthermore, an AAV-mediated knockdown of *Rpl3l* mRNA in the *mdx* mouse model of Duchenne muscular dystrophy and their wild type counterparts was reported to improve skeletal muscle function (Kao et al., 2021). This reduction in RPL3L expression also changed myofiber morphology after histological examination. However, skeletal muscle from our *Rpl3l* ^−/−^ mice did not display improved running endurance or altered atrophy after denervation surgery (Supplementary Figure S2), again suggesting that even in skeletal muscle RPL3L can be functionally compensated by RPL3.

We hypothesized that the developmental switching of RPL3 for RPL3L in the adult heart might underlie the known reduction in total protein synthesis as developmental growth dissipates. However, our ribosome profiling of wild type and *Rpl3l* ^*−/−*^ hearts suggested minimal changes, if any. Ribosome sequencing generally yields a substantial data set when an appropriate biologic pathway or regulator is altered (Doroudgar et al., 2019; van Heesch et al., 2019), yet we did not identify a significant difference in translated mRNAs between hearts of wild type versus *Rpl3l* ^*−/−*^ mice. It is thus unlikely that developmental switching observed for these two proteins from birth to early adulthood is required for changes in mRNA translation as the mouse heart matures. Indeed, a group of researchers that deleted the *Rpl22* gene in mice observed a compensatory increase of its paralog *Rpl22l1* that led to no substantial changes in translation phenotype (O’Leary et al., 2013). This is in contrast to a recent preprint that employed a ribosome pulldown assay coupled to nanopore sequencing in *Rpl3l* ^−/−^ hearts, which showed significant changes in mitochondrial pathway gene expression (Milenkovic et al., 2021). Again, we failed to identify significant alterations in mRNA levels of mitochondrial genes preferentially translated by wild type and *Rpl3l* ^*−/−*^ cardiac ribosomes.

Although there is relatively little known about the tissue-specific functions of ribosomes in cardiomyocytes, our results suggest that cardiac ribosomes can impact the aging mouse heart. Indeed, our most interesting finding was the age-related reduction in heart size in the *Rpl3l* ^−/−^ mice (Figures 5A, B). It is known that RPL3 occupies a position in the 60S ribosomal subunit that is very close to the peptidyl transferase center (Petrov et al., 2004), which fits with its known role in controlling translation efficiency. Indeed, certain mutations in the *Rpl3* gene in yeast can lead to three-to four-fold increases in frameshifting errors, impacting translational fidelity (Peltz et al., 1999). Recent data also show that the methylation of RPL3 can suppress translation elongation, increasing the time for nascent protein folding and increasing the fidelity of translation (Al-Hadid et al., 2016; Matsuura-Suzuki et al., 2022). This RPL3 methylation-induced translational control mechanism might be mirrored in how RPL3L impacts translation in avians and mammals, species that have more highly organized striated muscle cytoarchitecture than lower animals that do not express RPL3L. Moreover, striated muscle has an abundance of extremely large proteins that likely require longer periods of time to fold properly to ensure efficient structural packing in sarcomeres, and the RPL3L gene product might facilitate this process better than RPL3. Thus, our findings of significantly smaller hearts in 18 month old *Rpl3l* ^−/−^ mice may be the result of subtle differences in translation error rates and sarcomere protein packing that only become noticeable with age. Of note, mutations in RPL3L are known to also induce age-related cardiac dysfunction in humans (Thorolfsdottir et al., 2018). It would be interesting to model the known position of the methylation event in yeast RPL3 versus the position of key structural residues in RPL3L within the mammalian ribosome to better understand the positioning of these proteins near the peptidyltransferase center that might impact translation error correcting and rates of translation (Petrov et al., 2004).

In conclusion, RPL3L in the mouse can impact cardiac growth with aging and taken together with the observation that mutations in *RPL3L* in humans can cause severe cardiac abnormalities, it underscores the overall importance of this protein in striated muscle structure and function. However, future studies will be needed to fully understand how RPL3L impacts the function of the striated muscle ribosome in presumably mediating optimal sarcomere protein production and translational fidelity.

## Methods

### Mouse lines

*Rpl3l* gene-targeted mice were generated using two sgRNAs (target sequences: 5′-GTG​GGC​CTT​CTT​CTG​CCG​AA-3′ and 5′-TGG​CTG​AAC​ACG​CTG​TGT​AC-3′), according to the location and the on- and off-target scores from the web tool CRISPOR (Haeussler et al., 2016). The sgRNAs were synthesized *in vitro* using the MEGAshorscript T7 kit (ThermoFisher, #AM1354) and purified by the MEGAclear Kit (ThermoFisher, #AM1908), according to manufacturer’s instructions. The two sgRNAs (50 ng/μL each) and Cas9 protein (200 ng/μL, ThermoFisher, #A36499) were mixed at 37°C for 15 min to form the ribonucleoprotein complex and injected into the cytoplasm of one-cell-stage embryos of C57BL/6NTac genetic background (Taconic, model #B6) by piezo-driven microinjection (Scott and Hu., 2019). Injected embryos were immediately transferred into the oviductal ampulla of pseudopregnant CD-1 females (Charles River, strain 022). Live born pups were genotyped by PCR and Sanger sequencing (see Supplementary Tables S1 for primers). The *Rpl3l* targeted mouse line that was used here showed the effects of both sgRNAs in the same chromosome with deletions and frame-shift mutations in the fifth exon of *Rpl3l* that produced an out of frame mRNA and null allele.

RiboTag mice with Cre-mediated expression of 3 HA tags within the RPL22 protein were obtained from Jackson Laboratories (JAX; strain 011029, C57BL/6NJ background) (Sanz et al., 2009). These mice were crossed with *Myh6-*Cre transgenic mice (JAX strain 009074, C57BL/6 background), which have been previously described (Oka et al., 2006). The resultant offspring were bred to contain two targeted *Rpl3l* alleles or wild type alleles as a control. The previously described heart-specific MEK1 transgenic mice (JAX strain 010581, FVB background) expressed a constitutively active form of MEK1 protein such that the MEK1-ERK1/2 signaling pathway was activated, resulting in cardiac hypertrophy (Bueno et al., 2000). The previously described cardiac-specific calcineurin transgenic mouse line (JAX strain 009075, FVB background) expressed a constitutively active form of the calcineurin catalytic subunit that causes hypertrophy of the heart (Molkentin et al., 1998). To genotype the various mouse lines, tail samples were collected, and DNA was isolated using the Kingfisher Flex Purification system (ThermoFisher, #5400610) and PCR was conducted with the primers shown in Supplementary Tables S1. All mice were housed in a temperature-controlled environment with a 14/10 h light/dark cycle and *ad libitum* access to water and chow diet. All animal procedures were carried out in accordance with the Institutional Animal Care and Use Committee (IACUC)-approved protocol at CCHMC. Experimental groups were comprised of males and females in all groups and analyzed for sex-specific differences before combining. Only the Ribotag-aided ribosome immunohistochemistry was conducted in male mice because the αMHC-cre transgene is on the X chromosome.

### Quantitative PCR

To determine presence of selected mRNA transcripts in the heart, RNA was isolated from ventricular tissue using the RNeasy Fibrous Tissue Kit (Qiagen, #74704). cDNA was synthesized with SuperScript III First-Strand Synthesis SuperMix (Invitrogen, #18080400). To examine the potential transcript variants from the *Rpl3l* gene, forward primers to variants one and two and common reverse primer were generated (Supplementary Tables S1) and added to reaction mixture with Denville Choice-Taq DNA Polymerase (Thomas Scientific, #C775Y42) and 2 mM MgCl_2_. Resulting products were visualized by electrophoresis after loading on agarose gels containing ethidium bromide. For qPCR validation of microarray and ribosome sequencing data sets, 1 μg RNA was reverse transcribed to cDNA using the Verso cDNA synthesis kit (ThermoFisher, #AB1453B) according to manufacturers’ instructions. An equal mixture of oligo (dt) and random hexamers was used. To quantify gene expression changes, qPCR was performed using SsoAdvanced Universal SYBR^Ⓡ^ Green Supermix (Bio-Rad, #1725274) according to manufacturers’ instructions in a CFX96 PCR system (Bio-Rad, #1845096). An annealing temperature of 60°C was used for all primers and each reaction was subsequently subjected to melting curve analysis. All values were normalized to *Gapdh*. See Supplementary Tables S1 for primer sequences.

### Generation of RPL3 and RPL3L antibodies

Polyclonal antibodies against RPL3 and RPL3L were produced by YenZym Antibodies (Brisbane, CA, United States) in rabbits immunized against synthetic peptides for mouse RPL3 (amino acids 394–403) and mouse RPL3L (amino acids 395–407 of isoform 1) that were conjugated to Keyhole Limpet Hemocyanin (KLH). Two rabbits were immunized with each peptide. The rabbits were primed with the peptide-KLH conjugate in Complete Freund’s Adjuvant and boosted 4 times, 2 weeks apart with the same peptide-KLH conjugate in Incomplete Freund’s Adjuvant. The production bleed was taken 10 days after the final boost, and the antibody was affinity purified against the respective peptide.

### Echocardiography

Mice were anesthetized with 2% isoflurane in a 100% oxygen mix. They were secured to a temperature-controlled board equipped with electrocardiogram sensors (VisualSonics, Fujifilm) and heart rate was monitored throughout imaging. Echocardiography was performed with a VisualSonics Vevo 3100 (Fujifilm) using a MS550D probe at the parasternal short axis. Analysis was conducted in the VisualSonics software package.

### Transverse aortic constriction surgery

Two-month-old mice were anesthetized using 3% isoflurane and intubated with an 18-gauge catheter. The mice were continuously anesthetized during surgery with a mouse ventilator (SomnoSuite, TSE Systems) containing 1.7% isoflurane. A thoracotomy was performed, followed by isolation of the transverse aorta. A suture was tied around the transverse aorta and a 27-gauge needle, and the needle was then removed to generate constriction. The thoracic incision was sutured and was further sealed with GLUture (Zoetis, Butler Schein, #034418). After extubation, sustained-release buprenorphine (0.2 mg/kg) was injected subcutaneously in a volume of 0.03 mL to reduce pain. Mice were monitored daily after surgery. Sham surgery was performed in the same manner, except that the aorta was not constricted. Hearts were harvested 1 or 12 weeks after the surgeries.

### Hindlimb denervation surgery

To induce skeletal muscle atrophy, 2-month-old mice were subjected to a model of hind limb denervation for 10 days, as previously described (Hindi et al., 2018). Briefly, sciatic denervation of the right hind limb was performed under anesthesia with 2%–3% isoflurane. The right leg was shaved followed by generation of a 0.5 cm incision approximately 0.5 cm proximal to the knee on the lateral side of the leg. The muscles were separated, and the sciatic nerve was lifted with surgical forceps and a 5 mm piece was removed and the incision was closed with surgical staples. The left leg served as a non-denervated internal control by subjecting it to the same procedure, except that the sciatic nerve was left intact.

### Treadmill running

Mice at 2 months of age were challenged with enforced treadmill running using an Exer-3/6 treadmill (Columbus Instruments International) as described previously (Kwong et al., 2018). The treadmill had adjustable speed and inclination and was equipped with a small electric shock-delivering grid that was used in the training regimens for the running protocol. Electric shock intensity was set at five which generates a current of 0.97 mA and a voltage of 73, and the incline was set to 10%. For the short running protocol running, mice experienced acclimation at 0 m/min for 5 min and then moved to 20 m/min over 2 min and maintained at this speed for 20 min or until exhaustion. Exhaustion was defined as stay on the shock grid for more than 5 consecutive seconds, at which point the shock was automatically stopped. For the long running protocol running, mice experienced acclimation at 0 m/min for 5 min and then moved to 20 m/min over 30 min and maintained at this speed for 20 min or until exhaustion. Total running distance (m) was reported until mice reached either of the 2 time periods or became exhausted.

### Western blotting

Snap frozen heart, liver, and quadriceps tissues were homogenized using a handheld mechanical homogenizer (Omni Tissue Master 125). Halt protease and phosphatase inhibitor (ThermoFisher, #78442) was added to hypotonic lysis buffer (10 mM NaCl, 10 mM MgCl_2_, 10 mM Tris–HCl [pH 7.5], 0.5% NP-40). Protein content was quantified using a bicinchoninic acid colorimetric assay (ThermoFisher, #23227) and a BioTek Synergy 2 microplate reader (Agilent). Samples were resuspended in 5x Laemmli loading buffer and gels were loaded with 40 µg of protein per lane. After gel electrophoresis at 115 V, proteins were transferred to nitrocellulose membranes with a 0.2 μm pore size (Bio-Rad, #162-0112) at 85 V for 1.5 h using the standard wet transfer method. A Precision Plus Protein™ Dual Color Standard was utilized as the protein marker for all western blots (Bio-Rad, #1610374). Membranes were blocked in 5% milk in 1x TBST (20 mM Tris pH 7.5, 150 mM NaCl, 0.1% [w/v] Tween 20) for 1 h at room temperature with shaking before being incubated with primary antibody in blocking buffer overnight on a shaker at 4°C. Primary antibodies included RPL3 (YenZym custom at 1:500), RPL3L (YenZym custom at 1:500), GAPDH (Fitzgerald, #10R-G109A at 1:20000), RPLP0 (Proteintech, #11290-2-AP at 1:500), RPS6 (Cell Signaling, #2217 at 1:1,000), UBF (Santa Cruz, sc-13125 at 1:500), MEK1/2 (Cell Signaling, #8727 at 1:1,000), RPS11 (Bethyl Laboratories, #A303-936A at 1:500), and Calcineurin pan A (Milipore, #07-1491 at 1:1,000). The next day the membranes were washed three times for 10 min each in 1x TBST before applying appropriate Li-COR fluorescent secondary antibodies (IRDye 800CW Goat anti-Rabbit #926-32211 and IRDye 680LT Goat Anti-Mouse IgG1 #926-68050 both at 1:10000) in blocking buffer for 1 h at room temperature with shaking. Following three 5 min washes in 1x TBST, the blots were imaged on a Li-COR CLx Odyssey Imager.

### Mitochondrial oxygen consumption

Mitochondria were isolated from freshly excised hearts of 2-month-old-mice and oxygen consumption rate was measured using a Seahorse Extracellular Flux Analyzer (Agilent Technologies, XF24) as described previously (Huo et al., 2020). Briefly, 25 μg of isolated heart mitochondria were loaded onto XF24 cell culture microplates (Agilent Technologies, #102342-100) in KCl buffer (125 mM KCl, 20 mM HEPES, 2 mM MgCl_2_, 2 mM KH_2_PO_4_, and 40 µM EGTA, pH 7.2) containing 25 mM pyruvate, 10 mM malate and 5 mM ADP. Microplates were subjected to the XF Mito Stress Test Kit (Agilent Technologies, #103015-100) following the manufacturer’s standard protocol. After basal respiration was measured, the mitochondria were treated sequentially with 2 μM oligomycin, 5 μM carbonyl cyanide-4 (trifluoromethoxy) phenylhydrazone (FCCP), and 0.5 μM rotenone.

### Electron microscopy

Hearts of anesthetized mice were perfused with relaxing buffer (0.15% sucrose, 5% dextrose, 10 mM KCl in 1x PBS) for 3 min and then perfused with fixation buffer (1% paraformaldehyde, 2% glutaraldehyde in 100 mM sodium cacodylate pH 7.4) and fixed for 2 h. Samples were then cut into 1 mm cubes and further fixed overnight. Samples were then post-fixed in 1% OsO4 for 2 h and dehydrated with a graded acetone series. The samples were embedded in epon-812 resin. Ultrathin sections of all tissues were counterstained with uranyl acetate and lead salts. Images were obtained using a Hitachi 7600 transmission electron microscope connected to a digital camera (AMT Imaging, Biosprint16).

### Immunohistochemistry and histology

Hearts were excised and fixed in 10% zinc formalin overnight and then switched to 70% ethanol before processing and embedding in paraffin. Tissue was sectioned in 5 µm slices. Following dehydration of samples, antigen retrieval was performed with Tris-EDTA buffer (10 mM TrisHCl base, 1.3 mM EDTA, pH 9.0) in a vegetable steamer (Hamilton Beach, type VS04) for 20 min. After a 30 min cool-down period in tris-EDTA buffer at room temperature, sections were was three times in 1x PBS and then incubated in blocking solution (1% BSA, 0.1% cold water fish skin gelatin, 0.1% Tween 20 in 1x PBS) for 30 min. Primary antibodies were diluted in blocking buffer and then applied to the sections overnight at 4°C: HA (BioLegend #901513 at 1:500) and desmin (Abcam, ab15200 at 1:300). The next day, the sections were washed in 1x PBS and then secondary antibodies diluted in blocking buffer were applied for 1 h at room temperature: AlexaFluor Ms IgG1 488 (Invitrogen, A-21121 at 1:500) and AlexaFluor Rabbit 568 (Invitrogen, A-11011 at 1:500). Following three more washes in 1x PBS, 4′,6-diamidino-2-phenylindole (DAPI; 1:2,000) was applied to the samples for 10 min. Wheat germ agglutinin (WGA), Alexa fluor 594 conjugate (1:100) was also applied to a subset of samples for 1 h after a blocking step as described above, and then washed 3 times in 1x PBS before incubation with DAPI as described above. All slides had coverslips applied with ProLong Diamond mountant (ThermoFisher, #P36970). Samples were imaged using a Nikon A1R confocal microscope, and analysis of the images was conducted with Nikon Imaging Software (NIS) Elements Advanced Research software (Nikon). Visualization of collagen deposition was done with picrosirius red staining of cardiac histological sections following phosphomolybdic acid treatment as described previously (Dolber and Spach, 1987). Following staining, slides were mounted with Cytoseal (ThermoFisher, #831016) and given a coverslip. Slides were imaged using an Olympus BX51 microscope and NIS Elements Advanced Research software (Nikon).

### AAV generation

*Rpl3* and *Rpl3l* cDNA were generated from liver and heart RNA, respectively, using First-Strand Synthesis SuperMix (Invitrogen, #18080400). Firefly luciferase cDNA was cloned from the pGL3 luciferase reporter plasmid (Promega). The cDNAs were then cloned into the pAV-cTnT AAV expression vector (Vigene Biosciences) utilizing the NEBuilder HiFi DNA Assembly polymerase (New England BioLabs, #E2621L). For primers, see Supplementary Tables S1. The viruses were produced at the Howard Hughes Medical Institute Janelia Farms Viral Tools facility using the AAV9 capsid. Intrathoracic injection (50 µL volume, 28 G needle) using titers of 10^12^ viral genome copies was performed in 9-day-old mouse pups that were immobilized by cold anesthesia prior to injection (Wakimoto et al., 2016).

### Ribosome profiling

Hearts were perfused with ice-cold cycloheximide (150 μg/mL) at the time of collection, and then snap frozen in liquid nitrogen. The procedure used to isolate polysomes was based on that from Seimetz et al. (2020) (Seimetz et al., 2019). Briefly, sucrose gradients were prepared the evening before the experiment and allowed to settle at 4°C overnight. These consisted of 60%, 45%, 30%, and 15% sucrose solutions that were added to 4 mL thin-walled polypropylene tubes (Beckman Coulter, #328874) in the following amounts 0.6, 1.2, 1.2, and 0.6 mL, respectively. The sucrose solutions were made in a base solution of 10 mM Tris-HCl at pH 7.5, 100 mM KCl, and 5 mM MgCl_2_ in UltraPure DNase/RNase Free Distilled Water (ThermoFisher, #10-977-015).

Whole hearts (excluding atria) were powdered in a liquid nitrogen-cooled tissue pulverizer and then added to 2 mL glass Dounce homogenizers for 35 strokes in ice containing 1 mL of polysome lysis buffer (10 mM Tris-HCl at pH 7.5, 100 mM KCl, 5 mM MgCl_2_, 0.5% w/ve sodium deoxycholate, 1% v/v IGEPAL CA-630, 10 mM DTT, 150 μg/mL cycloheximide, 1U/μL SUPERase In RNase inhibitor [ThermoFisher, #AM2696], and 1X HALT protease inhibitor [ThermoFisher, #78439] in UltraPure DNase/RNase Free Distilled Water [ThermoFisher, #10-977-015]) and transferred to Eppendorf tubes and left to settle on ice for 20 min. Samples were spun at ×2,000 g at 4°C for 5 min. The supernatant was collected and subsequently spun at ×16,000 g at 4°C for 5 min. Approximately 700 µL of the final supernatant was layered on top of the sucrose columns and then loaded into a SW 60 Ti swinging bucket rotor (Beckman Coulter) and centrifuged at ×194,600 g for 125 min in a Optima XPN-90 ultracentrifuge (Beckman Coulter).

To isolate protein from the sucrose fractions, the remaining homogenate was pipetted off and discarded. Sucrose fractions were then collected in 300 µL increments and added to tubes containing 150 µL each of freshly prepared 4 mg/mL sodium deoxycholate and mixed well. Fifty microliters of freshly made 50% trichloroacetic acid in UltraPure DNase/RNase Free Distilled Water (ThermoFisher, #10-977-015) were added to each tube with thorough mixing. Protein was allowed to precipitate overnight at −20°C. The next day, samples were pelleted at ×20,000 g for 15 min at 4°C, supernatant discarded, and the pellet washed in 100% ethanol. The samples were pelleted again with the same parameters but washed in 75% ethanol. Following a third round of centrifugation, the supernatant was removed, and the pellet was air dried for 15 min. The pellets were re-suspended in 30 µL ×1 Laemmli buffer and heated at 95°C for 5 min. Samples were loaded on to SDS-PAGE gels and experiments proceeded according to the above western blotting protocol.

### Microarray and ribosome sequencing bioinformatic analyses

For microarray analysis, total RNA was extracted from frozen cardiac tissue using the Trizol method as described above and RNA quality was assessed using the 2100 Bioanalyzer (Agilent, #G2939A) at the Gene Expression Core Facility at Cincinnati Children’s Hospital. Microarray analysis of RNA samples from hearts of wild-type control and *Rpl3l* gene-deleted mice at 2–3 months of age was performed with Affymetrix Clariom S arrays (ThermoFisher, #902930, whole transcript expression array; GEO platform i.d: GPL23038). Bioinformatics analysis of resultant Expression Array feature intensity (CEL) files was carried out using the Transcriptome Analysis Console (TAC; Applied Biosystems; ver. 4.0.0.25), the Clariom_S_Mouse TAC Configuration file (ver. 4), and the iPathwayGuide (Advaita Bioinformatics) to determine differential gene expression profiles. Data were uploaded to the Genome Expression Omnibus (GEO) database (GSE216168).

Hearts of wildtype and *Rpl3l* ^*−/−*^ mice at 2–3 months of age were collected for ribosome sequencing after purification by sucrose gradient centrifugation as detailed above. RNA was prepared from these sucrose gradient fractions and then pooled, and then total RNA was isolated using a TriZol-based method. The quality of RNA was assessed using the 2100 Bioanalyzer (Agilent, #G2939A) at the Gene Expression Core at Cincinnati Children’s Hospital. Libraries were generated using the Ribo-zero + TruSeq Stranded pipeline followed by paired-end 100 bp sequencing conditions, with 40 million reads per sample. Bioinformatic analysis of data was carried out using Strand NGS software (ver. 2.6; Build: mouse mm10 [UCSC] using Ensembl transcript annotations) to identify differentially expressed genes. Gene clustering analysis of pathway gene expression groupings was performed with Strand NGS software. Fold change (FC) analysis was carried out using Strand NGS Software and identifies genes with ratio between experimental and control conditions, above an assigned threshold. Our analysis had two conditions; therefore, RNA from polysomal fractions isolated from wild type hearts were chosen as the reference condition against which the fold change for the *Rpl3l* ^*−/−*^ cardiac samples (experimental condition) was computed. As per the Strand NGS manual, the exact fold change is computed as follows: 1) For each gene and condition, the average of the normalized signal values (in log-scale) is calculated; 2) Log FC for a given gene between the two conditions is then the difference between their respective averages; 3) FC is then computed as (sign of Log FC) x 2ǀlogFCǀ; absolute FC is ǀFCǀ. Data were uploaded to the GEO database (GSE21586).

### Statistics

For all statistical analyses, values of *p* < 0.05 were considered significant. Where there was only one variable being tested between two groups, unpaired t-tests were conducted. For the qPCR data sets, unpaired t-tests were run on the 2^−ΔΔCT^ values while the relative RNA expression data points are shown. For analysis of groups of mice subjected to AAV gene transduction, one-way ANOVAs with Dunnett’s multiple comparisons tests were conducted. Where there were two factors (i.e., treatment and genotype) being tested a two-way ANOVA with a Sidak’s multiple comparisons test was conducted. All data are reported as mean ± standard error of the mean (SEM). Only the treadmill running data sets had outliers, as found by a Grubbs outlier test, and they were removed. Statistical analysis was conducted and graphs were generated with Graph Pad Prism (Dotmatics).

## Data Availability

The data sets presented in this study can be found in online repositories. The names of the repository/repositories and accession number(s) can be found in the article/Supplementary Material.
