# Supplementary material for: Rpl3l gene deletion in mice reduces heart weight over time
Source: Front Physiol. 2023 Jan 17;14:1054169. doi: 10.3389/fphys.2023.1054169 (PMC9886673; doi:10.3389/fphys.2023.1054169)
Supplement: Supplementary file 1 [file DataSheet2.PDF]

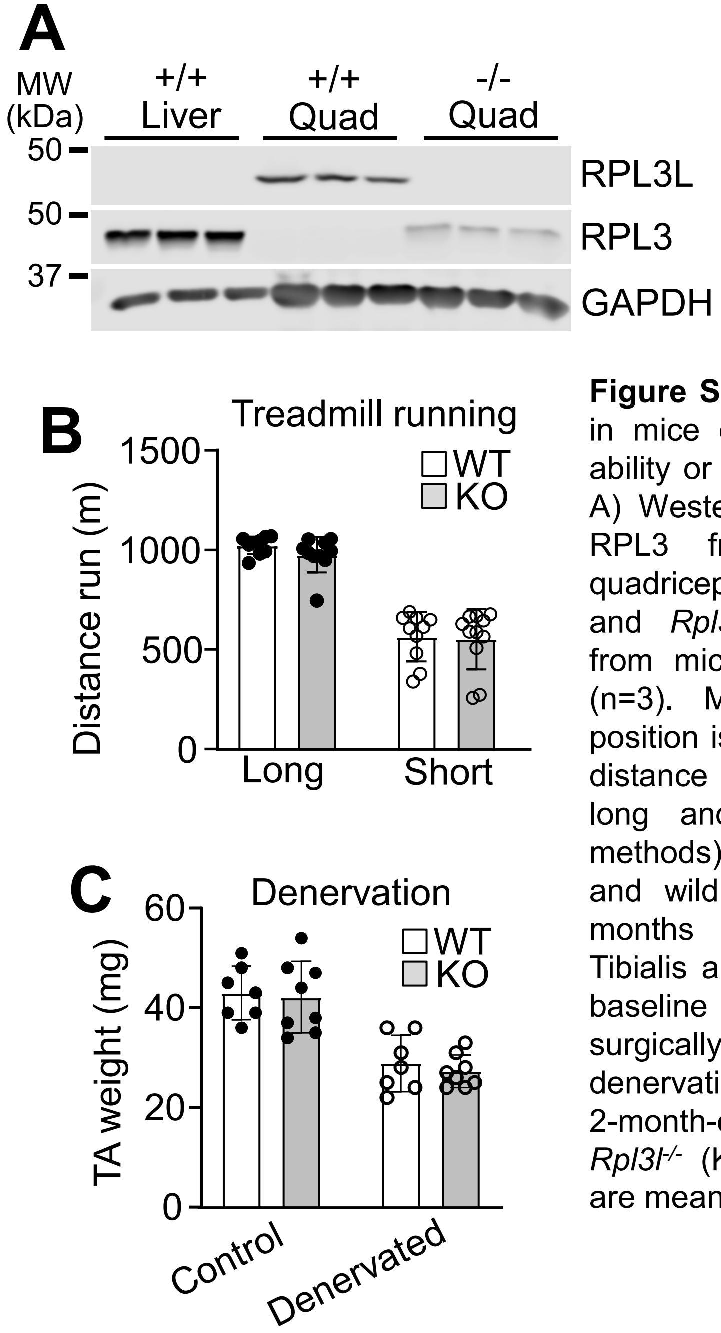

Figure S2: *Rpl3l* genetic deletion in mice does not affect running ability or skeletal muscle atrophy. A) Western blots of RPL3L and RPL3 from wild type (+/+) quadriceps and liver, as well as and *Rpl3l*$^{-/-}$ quadriceps muscle from mice at 2 months of age (n=3). Molecular weight (MW) position is shown in kDa. B) Total distance run on a treadmill in the long and short protocol (see methods) between *Rpl3l*$^{-/-}$ (KO) and wild type (WT) mice at 2 months of age (n=9-10). C) Tibialis anterior muscle weight at baseline (control) or after surgically induced atrophy by denervation for 10 days between 2-month-old wild type (WT) and *Rpl3l*$^{-/-}$ (KO) mice (n=7-8). Data are mean ± SEM.
