# Supplementary material for: Rpl3l gene deletion in mice reduces heart weight over time
Source: Front Physiol. 2023 Jan 17;14:1054169. doi: 10.3389/fphys.2023.1054169 (PMC9886673; doi:10.3389/fphys.2023.1054169)
Supplement: Supplementary file 2 [file Table1.pdf]

Table S1: Primers used for qPCR, PCR genotyping, and cloning

Primer	Usage	Sequence
Rpl3l TV1 Fwd	qPCR	5’-CTAACAGCCTTCCTGGGCTACAAG-3’
Rpl3l TV2 Fwd	qPCR	5’-TACCTGGTCCAGCTTGATGTTCAC-3’
Rpl3l common Rev	qPCR	5’-GTACAGGCACCTGCTTCTCCATC-3’
Rpl3 Fwd	qPCR	5’-GACAGGCCAGGATCTAAGGTG-3’
Rpl3 Rev	qPCR	5’-GGTCTCAACATATCCCACAATGC-3’
Mid1 Fwd	qPCR	5’-TGAAGACCAACAGTCAGCCG-3’
Mid1 Rev	qPCR	5’-TCGACAGTCAGGTTGTCGTG-3’
Inmt Fwd	qPCR	5’-CTGGAGGGAGACAGAAGCAG-3’
Inmt Rev	qPCR	5’-ACATCACACCTCAGGACACG-3’
Lce1e Fwd	qPCR	5’-TGTGCTACTCCTTATTTTGCTCC-3’
Lce1e Rev	qPCR	5’-GCACTTTGGAGGAGGCTGG-3’
Dusp10 Fwd	qPCR	5’-TCCCAGCCACTTCACATAGTC-3’
Dusp10 Rev	qPCR	5’-AGGGAGTTGTCACAGAGGTTTC-3’
Thbs1 Fwd	qPCR	5’-AGACCGGTTATATCAGAGTGGTG-3’
Thbs1 Rev	qPCR	5’-GCGCTGGTTATGATTGGCAG-3’
Mt2 Fwd	qPCR	5’-AATGTACTTCCTGCAAGAAAAGCTG-3’
Mt2 Rev	qPCR	5’-CAGCCCTGGGAGCACTTCG-3’
Nr4a3 Fwd	qPCR	5’-TTCTGACGGCCTCCATTGAC-3’
Nr4a3 Rev	qPCR	5’-AGCAGTGTTTGACCTGATGG-3’
Cdkn1a Fwd	qPCR	5’-ATCCAGACATTCAGAGCCACAG-3’
Cdkn1a Rev	qPCR	5’-CAAAGTTCCACCGTTCTCGG-3’
Ddx3y Fwd	qPCR	5’-CCGTGGACGTTCTAAAAGCAG-3’
Ddx3y Rev	qPCR	5’-AAACCTCCATAGCCACCTCC-3’
Ei2s3y Fwd	qPCR	5’-CAGTCAAGGCAGATTTGGG-3’
Ei2s3y Rev	qPCR	5’-GCCCCAACCAATTAAACGCC-3’
Ptgs Fwd	qPCR	5’-CCACCCTCTACAGCAGAACC-3’
Ptgs Rev	qPCR	5’-AATGTCCTCCTCTGTGAGGC-3’
Vaultrc5 Fwd	qPCR	5’-AGCTCAGCGGTTACTTCGAC-3’
Vaultrc5 Rev	qPCR	5’-ACGGGTTAGGTAAGTGGTTGG-3’
Mid1-ps Fwd	qPCR	5’-ATGCACAGGAGAGAAACGGG-3’
Mid1-ps Rev	qPCR	5’-TCGGTGAGATTTGCTCGTGG-3’
Nudt8 Fwd	qPCR	5’-GGTTGGGAGACACAAAGGG-3’
Nudt8 Rev	qPCR	5’-TGGTTGCCTTTTCCCGGTC-3’
Lypd8l Fwd	qPCR	5’-TGAGGTTACCAGCAGAGACCC-3’
Lypd8l Rev	qPCR	5’-TCCATTGGTCGAAATGCACG-3’
Rpl3l Fwd	PCR Genotyping	5’-TGGGGACTGTGAACTGACCTTAAT-3’
Rpl3l Rev	PCR Genotyping	5’-CATCAATAACCTCACTCTGGCTGAA-3’
RiboTag Fwd	PCR Genotyping	5’-GGGAGGCTTGCTGGATATG-3’
RiboTag Rev	PCR Genotyping	5’-TTTCCAGACACAGGCTAAGTA-3’
Cre Fwd	PCR Genotyping	5’-CGTACTGACGGTGGGAGAAT-3’
Cre Rev	PCR Genotyping	5’-TGCATGATCTCCGGTATTGA-3’

Mek1 Fwd	PCR Genotyping	5’-ACTTGCCCCTTGCTCCATAC-3’
Mek1 Rev	PCR Genotyping	5’-CTCATATGGAATGGACAGCCG-3’
Calcineurin Fwd	PCR Genotyping	5’-GTCTGACTAGGTGTCCTTCT-3’
Calcineurin Rev	PCR Genotyping	5’-CGTCCTCCTGCTGGTATAG-3’
Rpl3l Fwd	Cloning	5’-GAGATCTGCCGCCGCGATCGCATGTCCCACCGGAAG-3’
Rpl3l Rev	Cloning	5’-CTCGAGCGGCCGCGTACGCGTTGACAGAGAGAGTTG-3’
Rpl3 Fwd	Cloning	5’-GAGATCTGCCGCCGCGATCGCATGTCTCACAGGAAA-3’
Rpl3 Rev	Cloning	5’-CTCGAGCGGCCGCGTACGCGTGGGAAAACCAGTGTG-3’
Luciferase Fwd	Cloning	5’-GAGATCTGCCGCCGCGATCGCATGGAAGACGCAAA-3’
Luciferase Rev	Cloning	5’-CTCGAGCGGCCGCGTACGCGTTTACACGGCGATCTT-3’

Abbreviations: TV denotes transcript variants, Fwd denotes forward and Rev denotes reverse direction amplification.
