# Supplementary material for: Rpl3l gene deletion in mice reduces heart weight over time
Source: Front Physiol. 2023 Jan 17;14:1054169. doi: 10.3389/fphys.2023.1054169 (PMC9886673; doi:10.3389/fphys.2023.1054169)
Supplement: Supplementary file 3 [file Table2.pdf]

Table S2: Echocardiographic parameters in mice at baseline and after 1 week of TAC treatment

Rpl3l genotype:	+/+	-/-	+/+	-/-
	Baseline	Baseline	1 wk TAC	1 wk TAC
Number of mice	8	8	8	6
Heart Rate (bpm)	515.67 ± 27.15	499.97 ± 17.29	529.74 ± 28.99	537.29 ± 25.83
Left Ventricular Internal Diameter in Diastole (mm)	3.58 ± 0.11	3.64 ± 0.07	4.25 ± 0.17	4.12 ± 0.15
Average Left Ventricular Wall Thickness in Diastole (mm)	0.76 ± 0.02	0.75 ± 0.03	0.96 ± 0.03	0.95 ± 0.05
Fractional Shortening (%)	37.37 ± 1.89	42.04 ± 2.32	14.88 ± 1.91	17.74 ± 2.97

Table S3: Echocardiographic parameters in mice at baseline and after 12 weeks of TAC treatment

Rpl3l genotype:	+/+	-/-	+/+	-/-
	Baseline	Baseline	12 wk TAC	12 wk TAC
Number of mice	9	12	6	8
Heart Rate (bpm)	539.39 ± 15.01	497.80 ± 14.62	560.52 ± 42.93	575.08 ± 28.64
Left Ventricular Internal Diameter in Diastole (mm)	3.69 ± 0.06	3.87 ± 0.06	4.68 ± 0.23	4.64 ± 0.14
Average Left Ventricular Wall Thickness in Diastole (mm)	0.69 ± 0.02	0.71 ± 0.01	1.04 ± 0.05	0.93 ± 0.03
Fractional Shortening (%)	35.97 ± 2.69	36.27 ± 1.86	19.40 ± 2.42	14.97 ± 2.16
