# Supplementary material for: Rpl3l gene deletion in mice reduces heart weight over time
Source: Front Physiol. 2023 Jan 17;14:1054169. doi: 10.3389/fphys.2023.1054169 (PMC9886673; doi:10.3389/fphys.2023.1054169)
Supplement: Supplementary file 4 [file DataSheet1.PDF]

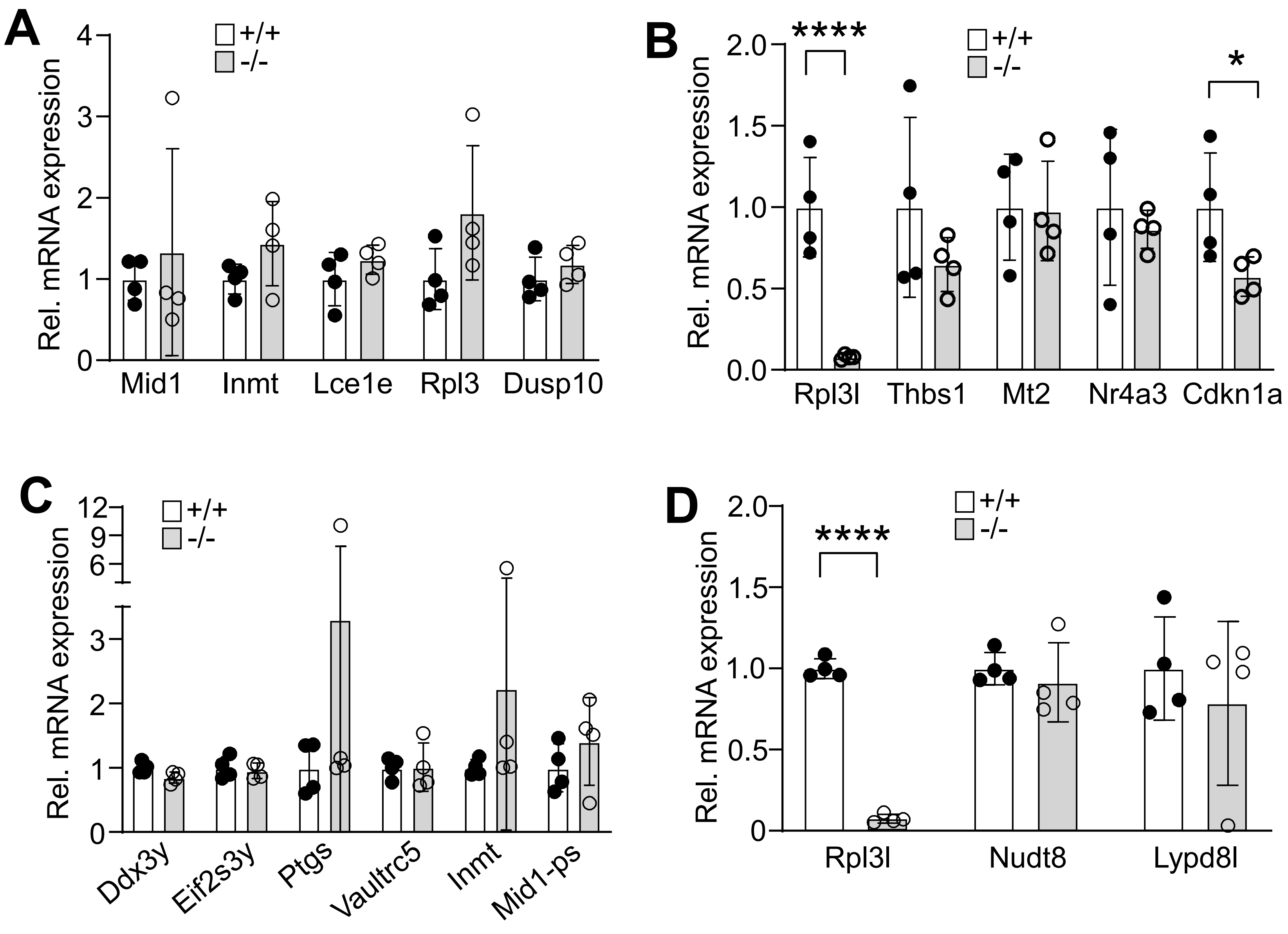

Figure S1: Quantitative PCR from cardiac mRNA to examine reproducibility of results from the microarray and ribosome sequencing results generated in wildtype versus *Rpl3l*$^{-/-}$ mice at 2-3 months of age. A) qPCR for the indicated genes that represent the 5 most upregulated and, B) the 5 most downregulated transcripts from the microarray dataset (**** = $p < 0.0001$, * = $p < 0.05$). Only Rpl3l and Cdkn1a validated, while the remaining transcripts were not altered and represent false positives in the Affymetrix array data. C) All upregulated and D) downregulated mRNAs from the ribosome sequencing dataset (**** = $p < 0.0001$). Data are mean ± SEM. Only Rpl3l validated as significantly changed, and all others were false positives from the ribosome sequencing procedure.
